# Dynamic predictive templates in perception

**DOI:** 10.1016/j.cub.2024.07.087

**Published:** 2024-08-21

**Authors:** Veith Weilnhammer, Yuki Murai, David Whitney

**Affiliations:** 1 Helen Wills Neuroscience Institute, University of California, Berkeley, Berkeley, CA 94720, USA; 2 Center for Information and Neural Networks, National Institute of Information and Communications Technology, Osaka 565-0871, Japan; 3 Graduate School of Frontier Biosciences, Osaka University, Osaka 565-0871, Japan; 4 X (formerly Twitter): @v_weilnhammer; 5 Lead contact

## Abstract

Hallucinations are vivid and transient experiences of objects, such as images or sounds, that occur in the absence of a corresponding stimulus.^1-9^ To understand the neurocomputational mechanisms of hallucinations, cognitive neuroscience has focused on experiments that induce false alarms (FAs) in healthy participants,^1-5,9^ psychosis-prone individuals,^1,3,4^ and patients diagnosed with schizophrenia.^5^ FAs occur when participants make decisions about difficult-to-detect stimuli and indicate the presence of a signal that was, in fact, not presented. Since FAs are, at heart, reports, they must meet two criteria to serve as an experimental proxy for hallucinations: first, FAs should reflect perceptual states that are characterized by specific contents^10-12^ (criterion 1). Second, FAs should occur on a timescale compatible with the temporal dynamics of hallucinations^13,14^ (criterion 2). In this work, we combined a classification image approach^15^ with hidden Markov models^16^ to show that FAs can match the perceptual and temporal characteristics of hallucinations. We asked healthy human participants to discriminate visual stimuli from noise and found that FAs were more likely to occur during an internal mode of sensory processing, a minute-long state of the brain during which perception is strongly biased toward previous experiences^17^ (serial dependency). Our results suggest that hallucinations are driven by dynamic predictive templates that transform noise into transient, coherent, and meaningful perceptual experiences.

## In brief

Weilnhammer et al. show that false alarms are more likely to occur during the internal mode, a minute-long state of the brain during which perception is strongly biased toward previous experiences.

## RESULTS

### Criterion 1: The perceptual quality of FAs

According to Bayes theorem, alarms (*P*(*signal*∣*input*)) become likely when people expect to encounter a signal (*P*(*signal*), prior), and when the features of a noisy input are compatible with the expected signal (*P*(*input*∣*signal*), likelihood). In natural environments, where the recent past is a predictor of the near future, previous stimuli may induce expectations about what is likely to be perceived next.^17,18^ This generates predictive templates that integrate experiences over time^19^ (*P*(*signal*∣*input*) = *P*(*signal*) × *P*(*input*∣*signal*)), giving rise to the phenomenon of perceptual serial dependence.^20-24^

Predictive templates may improve the interpretation of noisy but predictable sensory inputs.^19,25^ When overly strong, however, predictive templates may cause people to experience spurious signals in noise (*P*(*signal*∣*noise*)). Indeed, false alarms (FAs) can be induced via cross-modal conditioning^1,3-5^ and manipulations of signal probability.^9^ A number of empirical findings indicate that such conditioned FAs may provide a window into the neural mechanisms of hallucinations: conditioned FAs are more frequent in hallucination-prone individuals^1-4,9^ and patients diagnosed with schizophrenia.^5^ Moreover, they can be triggered by dopamine,^9^ an established molecular endophenotype of schizophrenia,^26^ as well as by ketamine,^9^ an NMDA-receptor antagonist that elicits psychotic symptoms.^27^

**Figure 1.**
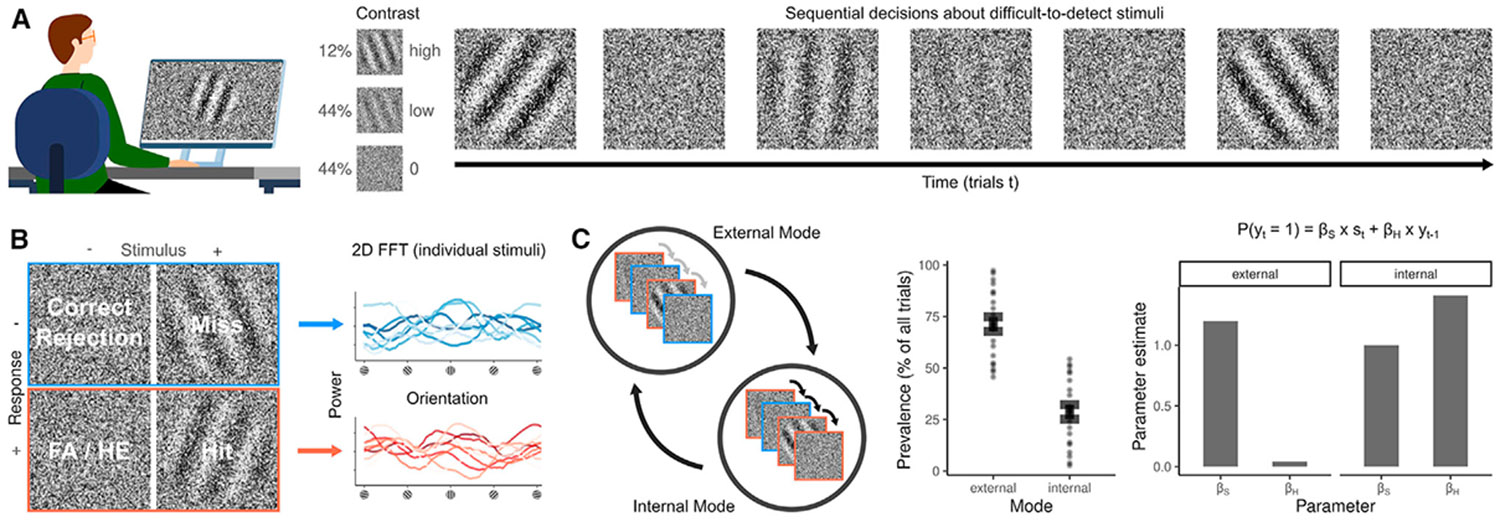
False alarms as an experimental proxy for hallucinations (A) Paradigm. Participants were instructed to report whether they perceived (close-to-) vertical gratings embedded in noise (left). At stimulus trials, gratings were presented either at low or at high contrast. At no-stimulus trials, no grating was presented (contrast = 0). We reasoned that alarms would be more frequent when participants expect to experience a signal and when the sensory noise is compatible with the predicted signal ((*P*(*signal*∣*noise*) = *P*(*signal*) × *P*(*noise*∣*signal*), top). We induced signal expectations *P*(*signal*) by presenting stimuli in sequence (right). We hypothesized that such a sequence would induce false alarms (FAs) via the phenomenon of serial dependence. Serial dependencies bias perception toward preceding experiences, causing alarms to be more likely to be followed by alarms and rejections more likely to be followed by rejections. Since serial dependencies are known to be stronger after confident experiences, we predicted that FAs would be more frequent after high-contrast stimuli. In the example shown on the right, our hypothesis therefore is that FAs would be most likely at the 2nd and 7th trial. (B) Predictive perceptual templates. To compute the compatibility of the white noise with the expected signal (*P*(*noise*∣*signal*)), we subtracted the power-by-orientation distribution of the random white noise images at rejection trials (blue) from the power-by-orientation distribution of the random noise images at alarm trials (red). The right panel shows the power-by-orientation distributions of 20 example stimuli that occurred at alarm and rejection trials. (C) External and internal modes in perception. Perception is known to slowly alternate between two opposing modes (left): during the external mode, perception is dominated by incoming stimuli, with weak serial dependencies between subsequent trials (gray arrows). By contrast, during the internal mode, perception is strongly biased by preceding experiences (strong serial dependencies, black arrows). The examples depicted within the circles illustrate the effect of external and internal mode on perception: in the external mode, perception dissociates from predictions that stem from serial dependencies, such that alarms (red) and rejections (blue) are equally likely to occur after high-contrast stimuli. In the internal mode, perception is strongly biased toward previous experiences, causing alarms (red) to be more frequent after high-contrast stimuli. Between-mode alternations can be discovered by hidden Markov models (HMMs). HMMs describe alternations between two independent general linear models (GLMs), each of which predicts perceptual experiences $y_{t}$ via weights assigned to the stimulus $\beta_{S}\times s_{t}$ at the current trial and the experience made at the preceding trial $\beta_{H}\times y_{t-1}$. In this experiment, the GLM-HMM discovered an external mode (71.3% ± 3.5% of trials), during which $\beta_{S}$ is high and $\beta_{H}$ is low (middle and right panels), and an internal mode (28.7% ± 3.5% of trials), during which $\beta_{S}$ is low and $\beta_{H}$ is high.

**Figure 2.**
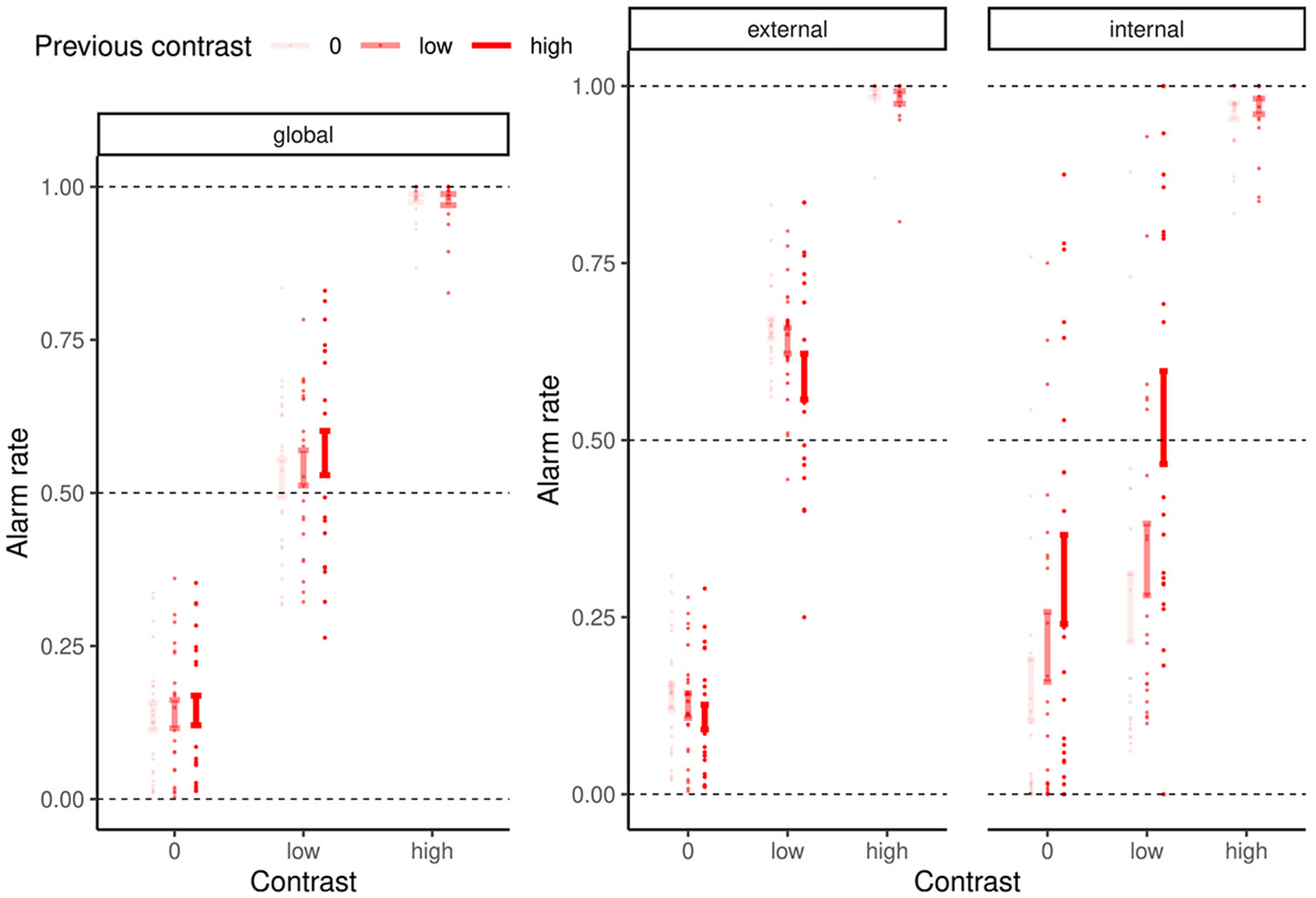
The effect of serial dependencies on alarms and rejections depends on mode The global alarm rate correlated positively with the contrast of stimuli presented at the preceding trial (0.11 ± 0.03, z = 4.33, *p* < 0.001). This suggests that serial dependencies contribute to FAs. When separating our data in episodes of external and internal modes, we found positive serial dependencies only during the internal mode (0.96 ± 0.06, z = 16.96, *p* < 0.001). During the external mode, by contrast, we observed a negative correlation between alarm rate and preceding stimulus contrast (−0.19 ± 0.03, z = −6.31, *p* < 0.001). Error bars indicate SEM. See also Figures S1 and S2.

The critical question is whether FAs are driven solely by signal expectations (*P*(*signal*), prior), in which case they may just be behavioral responses without any perceptual quality, or if they also depend on the compatibility of the noise with the expected signal (*P*(*noise*∣*signal*), likelihood). The latter case would indicate that FAs are accompanied by perceptual experiences with a specific content. To test the contribution of prior and likelihood to FAs, we analyzed data from 22 participants who judged whether close-to-vertical gratings (signals) were present (alarms) or absent (rejections) in white noise images (Figure 1A). The alarm rate depended on three factors: the contrast of the signal at the current trial (4.38 ± 0.07, z = 60.76, *p* < 0.001), the contrast of the signal at the preceding trial (0.11 ± 0.03, z = 4.33, *p* < 0.001; Figure 2), and the power of the current noise image at close-to-vertical orientations (60° to 120°), relative to the total power over all orientations (0.1 ± 0.01, z = 8.97, *p* < 0.001; Figure 1B).

**Figure 3.**
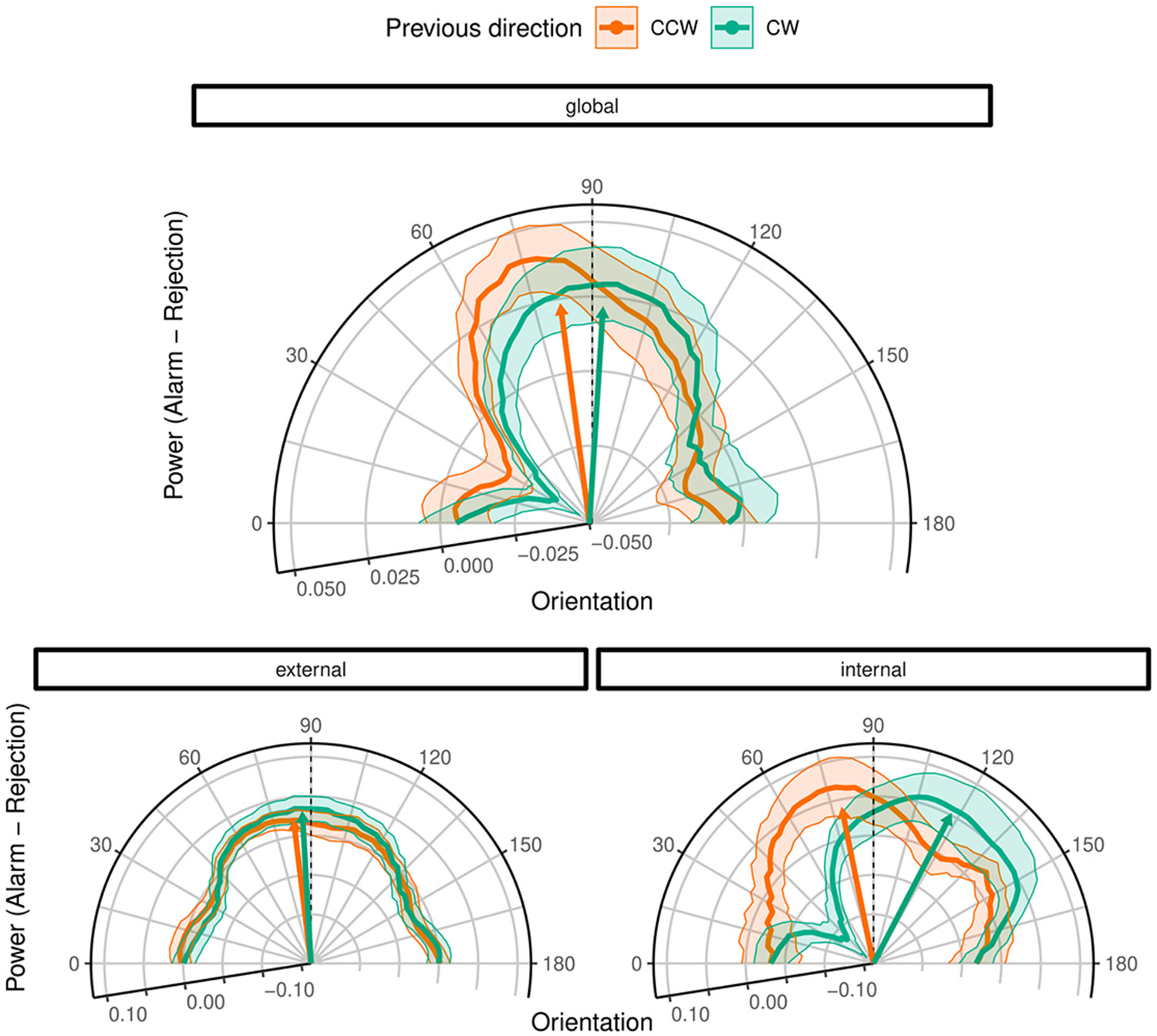
The effect of predictive templates on alarms and rejections depends on mode Globally, the classification images computed for alarms (power-by-orientation distribution at alarm trials relative to the power-by-orientation distribution at rejection trials) revealed a peak around 90°. This corresponds to the average orientation of the signal (0.1 ± 0.02, T(1.37 × 10^4^) = 7.24, *p* < 0.001). During the internal mode, we found a shift of the close-to-vertical noise power peak toward the orientation of the preceding stimulus (19.87 ± 6.73, T(20.28) = 2.95, *p* = 0.007; classification images after clockwise [CW] stimuli in green and counter-clockwise [CCW] in orange; dots indicate position of the average vector; orientations greater than 90° indicate CW displacement). This shift was not observed during the external mode (−3.67 ± 6.27, T(20.15) = −0.59, *p* = 0.56). The shaded area corresponds to the SEM. See also Figures S1 and S2.

This confirms that FAs were driven by predictive templates: FAs were more likely when the stimulus presented at the preceding trial induced a strong signal expectation (*P*(*signal*)) and when the sensory noise at the current trial matched the features of the expected signal (*P*(*noise*∣*signal*)). To visualize these predictive templates, we subtracted the noise power at rejection trials from the noise power at alarm trials (Figure 3). The resulting classification images^15^ revealed a noise power peak at close-to-vertical orientations (0.1 ± 0.02, T(1.37 × 10^4^) = 7.24, *p* < 0.001) that matched the average orientation of the signals.

If FAs were simply lapses or biases in response behavior, one would expect a flat classification image that mirrors the overall flat power-by-orientation distribution in the white noise stimuli used in our experiment. This was clearly not the case: FAs occurred more frequently at noise stimuli that matched a feature of the signal (high power at close-to-vertical orientations; Figure 3). Critically, observers learned the features of the average signal from the experiences they made throughout the experiment.

Our findings therefore support criterion 1: at the time of FAs, observers perceived specific contents—in this case, gratings with a close-to-vertical orientation. The hallucinated content was determined by predictive templates that are generated by the sequence of preceding experiences^19-24^ (*P*(*signal*∣*noise*) = *P*(*signal*) × *P*(*noise*∣*signal*)).

### Criterion 2: The temporal dynamics of predictive templates

Hallucinations are transient, coherent, and often meaningful experiences that unfold over time.^10-14^ A valid experimental proxy for hallucinations should therefore fluctuate at a timescale compatible with the duration of hallucinatory experiences. In the case of FAs, this means that the underlying predictive templates should be dynamic and generate recurrent and transient intervals during which perception is strongly biased toward previous experiences.

To assess the temporal dynamics of the predictive templates, we estimated hidden Markov models (HMMs) that inferred transitions between two latent states, each linked to a general linear model (GLM) that predicted trial-wise responses $y_{t}$ (alarms versus rejections) from the signal contrast at the current trial ($\beta_{s}\times s_{t}$) and the response at the preceding trial ($\beta_{H}\times y_{t-1}$; Figure 1C). In line with previous results,^16,17^ the HMMs revealed slow alternations between an external mode, where perception closely followed the external stimulus ($\beta_{S}>\beta_{H}$), and an internal mode, where perception was strongly biased by preceding experiences ($\beta_{S}<\beta_{H}$; Figure 1C).

Bayesian model comparison indicated that the two-state GLM provided a more parsimonious description of the data than the one-state control model (reduction in BIC: $\delta_{BIC}=-1.12\times 10^{3}$; see Figure S1 for control analyses on randomly permuted data, for which the advantage of the two-state GLM was lost).

Participants spent 28.7% ± 3.5% of their time in internal mode, with alternation between modes occurring in intervals of 59.1 ± 8.8 trials, corresponding to 118.21 ± 17.6 s. External and internal mode fluctuated spontaneously and were only marginally modulated by the contrast of the signal grating (Figure S2). The GLMs that defined the external and internal mode were independent from the orientation of the signal, the contrast of the signal at the preceding trial, and the power-by-orientation distribution of the noise. Any between-mode difference in the classification images was therefore indicative of dynamic changes in the underlying predictive templates.

We found that the internal mode increased the rate of FAs relative to the external mode (0.1 ± 0.02, T(109) = 4.61, *p* < 0.001; Figure 2). In the internal mode, perception depended more on the contrast of the stimulus presented at the preceding trial (1 ± 0.06, z = 16.86, *p* < 0.001) and less on contrast of the stimulus presented at the current trial (−1.16 ± 0.15, z = −7.64, *p* < 0.001). The effect of signal expectations (*P*(*signal*)) on perception was thus exaggerated during the internal mode.

Alarms during the internal mode were associated with a close-to-vertical noise-power peak that was shifted toward the orientation of the stimulus presented at the preceding trial (19.87 ± 6.73, T(20.28) = 2.95, *p* = 0.007; Figure 3). This shift was not observed during the external mode (−3.67 ± 6.27, T(20.15) = −0.59, *p* = 0.56). This suggests that the predictive templates and the associated bias in the content of perception toward previous experiences were particularly strong during the internal mode.

Our findings therefore confirmed criterion 2: the predictive templates that drive FAs are dynamic and fluctuate according to two opposing external and internal modes of perception. Alternations between modes occurred on the order of minutes and were thus compatible with the temporal duration of hallucinations. During the internal mode, FAs were associated with hallucinated contents that were skewed toward previous experiences.

## DISCUSSION

Our results indicate that the FAs observed here are dynamic perceptual phenomena, which are characterized by specific contents and are driven by dynamic predictive templates. These templates fluctuate in intervals of several minutes and are particularly strong during internal modes of sensory processing. This confirms that FAs induced by signal-detection experiments can match the perceptual^10-12^ and temporal^13,14^ quality of hallucinations.

Our findings suggest that FAs, and potentially hallucinations, are linked to alternations between external and internal modes of perception. External and internal modes have repeatedly been observed in mice,^17,28^ healthy human participants,^17^ and patients diagnosed with schizophrenia.^16^ We found that the internal mode not only increased the rate of FAs, but also enhanced the extent to which the content perceived at the time of FAs was skewed toward previous experiences.

This finding points to a tentative mechanism for how hallucinations emerge, persist, and end: during the internal mode, a hallucination may emerge as an FA that occurs when (1) a specific context induces signal expectations (e.g., hearing the neighbors voice in one’s apartment, *P*(*signal*)) and (2) the sensory input is compatible with the average features of the expected signal (e.g., construction noise that matches the features of human speech, *P*(*input*∣*signal*)). The internal mode enhances the effect of preceding experiences on perception and may allow the hallucinated content to persist over time as a coherent experience. A transition from internal to external mode greatly reduces the strength of the predictive template in which the hallucination unfolds, causing the end of the experience. Future research could test this hypothesis by testing whether FAs, and ultimately the hallucinations experienced by people living with psychotic symptoms, can be mitigated by interventions that disrupt the internal mode.

During the internal mode, the average vectors of the classification images were displaced by 20.18° ± 3.12° from vertical (Figure 3). The direction of the vectors depended on the predictive template, i.e., the orientation of the stimulus presented at the preceding trial. Interestingly, the displacement of the classification vectors from vertical was larger than the orientation of the high-contrast gratings (−10° and 10° from vertical; Figure 1A), which were most effective at inducing FAs during the internal mode (Figure 2). Since the high-contrast gratings stood out from the other stimuli in terms of contrast and orientation (low-contrast gratings were presented at orientations ranging from −3 to 3; Figures 1A and 1B), one may suspect that the perceived orientation of the high-contrast gratings exceeded their veridical orientation. An alternative explanation may be that FA experiences exaggerated the features of the inducer, just as hallucinations often portray an exaggerated version of everyday stimuli (such as hallucinated speech that stands out in terms of tone, pitch, or extreme content^10-12^).

Our findings suggest a key role of serial dependence^20-23^ and predictive templates^24^ for how hallucinations emerge and persist over time. This view overlaps with the so-called strong prior account of schizophrenia,^8^ where psychotic symptoms, and in particular hallucinations, are thought to occur due to an exaggerated effect of prior predictions on perception.^1-5,9^ At the same time, they seem at odds with the observation that priors are less influential in psychosis-prone individuals^29^ and patients diagnosed with schizophrenia^30^ (the weak prior account of schizophrenia^6^). So far, attempts to reconcile this disparate set of findings have argued that priors may be either weak or strong depending on the stage of psychotic illness^8^ (weak priors in early stages and strong priors in later stages) or the cognitive hierarchy^8^ (weak priors at the perceptual level and strong priors at the cognitive level).

The fact that FAs, and potentially psychotic symptoms such as hallucinations, are tied to recurrent alternations between external and internal modes of perception may provide an alternative explanation for the apparent discrepancy between strong and weak priors: psychosis-prone individuals and patients diagnosed with schizophrenia may share a general propensity toward the external mode, which reduces the effects of priors on perception^29^ and drives erratic responses to ambiguous sensory information.^16^ During the internal mode, psychosis-prone individuals and patients diagnosed with schizophrenia may have stronger predictive templates that trigger and shape the content of hallucinations. The combination of classification images and HMMs provides a novel analytical framework to test this hypothesis, may help to advance our theoretical understanding of schizophrenia, and can pave the way toward innovations in mental health that benefit people living with psychotic symptoms.

## STAR★METHODS

### RESOURCE AVAILABILITY

#### Lead contact

Further information and requests for resources should be directed to and will be fulfilled by the lead contact, Veith Weilnhammer (veith.weilnhammer@gmail.com).

#### Materials availability

This study did not generate new unique reagents.

#### Data and code availability

- All data have been deposited at https://github.com/veithweilnhammer/predictive_templates and are publicly available as of the date of publication. DOIs are listed in the key resources table.
- All original code has been deposited at https://github.com/veithweilnhammer/predictive_templates and is publicly available as of the date of publication. DOIs are listed in the key resources table.
- Any additional information required to reanalyze the data reported in this paper is available from the lead contact upon request.

### EXPERIMENTAL MODEL AND SUBJECT DETAILS

Twenty-two healthy volunteers (12 female, aged 18-26) took part in the experiment. All participants reported having normal or corrected-to-normal vision. The experiments were conducted following the guidelines of the institutional review boards of the University of California at Berkeley. We obtained written informed consent from all participants prior to the start of the experiment.

### METHOD DETAILS

#### Apparatus

The participants viewed visual stimuli on a gamma-corrected CRT monitor (Sony Trinitron Multiscan G520, 1024x768 pixels, 100 Hz refresh rate, Konica Minolta LS-110 luminometer for gamma correction) at a viewing distance of 57.3 cm, with head stabilization provided by a chin rest.

#### Stimuli and procedure

All stimuli were presented using MATLAB (MathWorks, R2017a) and the Psychophysics Toolbox. In 44% of the trials, we presented low-contrast near-to-vertical Gabor patches which were embedded in static white noise. In another 44% of the trials, we presented only static white noise. The remaining 12% of the trials featured a high-contrast Gabor patch (40% Michelson contrast) embedded in white noise. This high-contrast inducer was introduce to boost the phenomenon of serial dependency. The inducers were oriented 10° clockwise or counterclockwise relative to vertical, and presented in intervals randomized between 4 and 10 trials. Stimuli were displayed for 500 ms, with a maximum white noise contrast of 60%. To reduce potential luminance aftereffects, the signal was masked by low-pass filtered luminance contrast noise (100% contrast) for 500 ms, and the Gabor phase was randomized across trials.

Visual stimuli measured 14 × 14 degrees of visual angle (d.v.a.), with Gabor patches confined to a Gaussian contrast envelope with a 3 d.v.a. standard deviation. The spatial frequency of the Gabor patches was 0.5 cycles per degree (cpd), with orientation randomized within ±3° relative to vertical for low-contrast gratings. High-contrast gratings were presented at ±10°. Participants were tasked to judge the presence or absence of the near-vertical Gabor in the noisy image. We adjusted the signal contrast on a participant-by-participant basis to achieve a d’ of approximately 1.5 using data from preliminary experiments. Each session consisted of 100 trials, with inter-trial intervals randomized between 800 and 1200 ms. Participants completed 15-25 sessions in about 2 h.

The raw data were published in a previous report on serial dependencies in perception.^15^ The re-analysis presented here focuses on a different question (the perceptual and temporal quality of FAs as a proxy for hallucinations) and uses a different analytical approach (HMM-GLMs).

### QUANTIFICATION AND STATISTICAL ANALYSIS

Responses were categorized into one of four stimulus-response types: Hits (alarms on stimulus trials), correct rejections (rejections on no-stimulus trials), FAs (alarms on no-stimulus trials), and misses (rejections on stimulus trials). We employed standard logistic and linear regression using R-packages lmer, glmer, and afex (see key resources table, Method section). Unless otherwise specified, errorbars denote mean ± standard error of the mean (SEM).

#### Classification images

Following previous approaches,^15^ we filtered each static white noise image with the Gaussian stimulus envelope, applied fast 2-D Fourier transformation, and extracted the power at the Gabor patch’s spatial frequency, separately for orientations from 0 to 180° in 3° intervals. For every image, we computed the average power between 60 and 120°, relative to the overall power from complete orientation range. To visualize the classification images, we subtracted the average power-by-orientation at rejection trials from the average power-by-orientation at alarm trials. To examine serial dependence, the same analysis was conducted separately for trials following clockwise (CW) and counter-clockwise (CCW) inducers. In each condition and participant, we determined the average vector of the classification image by computing the centroid of a polygon composed of vector endpoints in polar coordinates.

#### Hidden Markov modeling

We used General Linear Models (GLM) to predict the response $y_{t}$ (0: rejection: 1: alarm) from the contrast at the current trial ($s_{t}$: zero contrast, low contrast, high contrast) and the response at the preceding trial $y_{t-1}$:

(Equation 1) $$P(y_{t}=1|x_{t})=\frac{1}{1+e^{-x_{t}\times w}}$$

(Equation 2) $$x_{t}\times w=s_{t}\times \beta_{s}+y_{t-1}\times \beta_{H}$$

We then used Hidden Markov Models (HMMs, as implemented in the *SSM: Bayesian learning and inference for state space models* python library, https://github.com/lindermanlab/ssm) to model transitions between two latent states k and j. At each trial, the HMM predicted the latent state $z_{t}$ was k or j, with alternation between k and j that were governed by a 2 x 2 transition matrix A:

Each latent state was linked to an independent GLM that predicted $y_{t}$ from $s_{t}$ and $y_{t-1}$ based on the weights $w_{j∕k}$:

(Equation 3) $$P(y_{t}=1|x_{t},z_{t})=\frac{1}{1+e^{-x_{t}\times w_{k∕j}}}$$

(Equation 4) $$x_{t}\times w_{k∕j}=s_{t}\times \beta_{s,k∕j}+y_{t-1}\times \beta_{p,k∕j}$$

For both states, parameters were initialized at the regression weights of a one-state control GLM. To compare the performance of the two-state GLM relative to the one-state control GLM, we computed the difference in Bayesian Information Criterion (BIC) between the two models. The SSM hyperparameters were defined as follows: $\sigma^{2}=10$ (variance over the GLM weights $w_{k∕j}$, and α=1 (Dirichlet prior over the transition matrix A) was set to 1. The latent states k and j were linked to mode by comparing $\beta_{s}$ and $\beta_{H}$ (external mode: $\beta_{s}>\beta_{H}$, internal mode: $\beta_{H}>\beta_{s}$). We labeled trial as external when $P(z_{t}=external)=1-P(z_{t}=internal)>0.5$.

## Supplementary Material

Supplemental Material

Supplemental information can be found online at 10.1016/j.cub.2024.07.087.

## Figures and Tables

**Table:** KEY RESOURCES TABLE

REAGENT or RESOURCE	SOURCE	IDENTIFIER
Deposited data		
Analyzed data & custom code	https://github.com/veithweilnhammer/predictive_templates/	10.5281/zenodo.13306545
Software and algorithms		
Python 3	http://www.python.org/	RRID: SCR_008394
Jupyter Notebook	https://jupyter.org/	RRID: SCR_018315
numpy	http://www.numpy.org	RRID: SCR_008633
pandas	https://pandas.pydata.org	RRID: SCR_018214
SSM	https://github.com/lindermanlab/ssm	N/A
MATLAB	https://www.mathworks.com/	RRID: SCR_001622
Psychtoolbox 3	http://psychtoolbox.org/	RRID: SCR_002881
R	http://www.r-project.org/	RRID: SCR_001905
RStudio	https://www.rstudio.com/	RRID: SCR_000432
lme4, afex, ggplot2, ggridges, gridExtra, tidyr, plyr	http://cran.r-project.org/	RRID: SCR_003005

## Highlights

- Perception alternates between external and internal modes
- In the external mode, perception follows the sensory signals
- In the internal mode, perception is determined by serial dependencies
- False alarms are more likely to occur during the internal mode
