# Supplementary material for: Dynamic predictive templates in perception
Source: Curr Biol. Author manuscript; Available in PMC 2026 Mar 20. (PMC13002333; doi:10.1016/j.cub.2024.07.087)
Supplement: Supplemental Material [file NIHMS2143895-supplement-Supplemental_Material.pdf]

Current Biology, Volume *34*

Supplemental Information

Dynamic predictive templates in perception

Veith Weilnhammer, Yuki Murai, and David Whitney

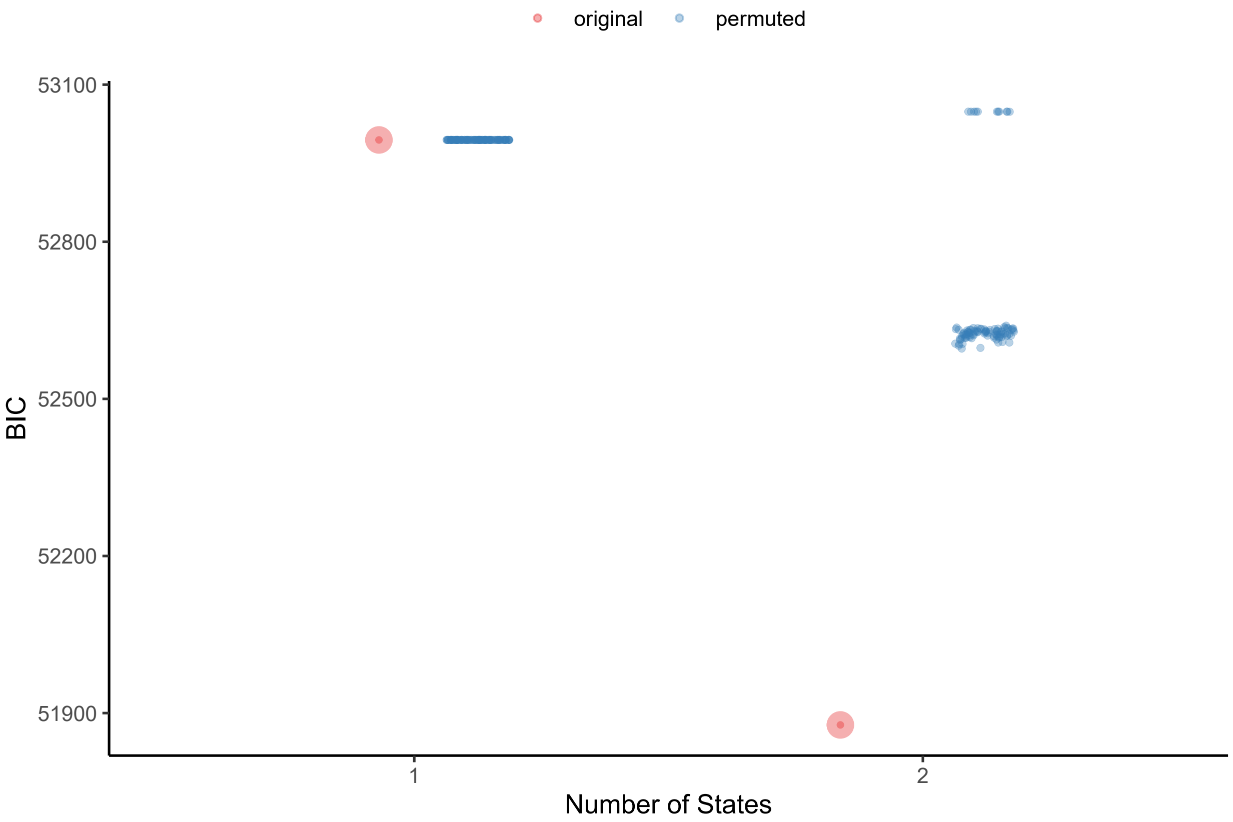

Figure S1. Model comparison between the two state GLM-HMM and a one-state control model. Related to Figure 2 and 3. We used Bayesian model comparison to evaluate whether the two-state HMM-GLM (number of parameters: 8) provided a more parsimonious explanation of our data in comparison to the one-state control model (number of parameters: 2). To compare the two models, we computed the difference in BIC $= \log(N) \times k - 2 \times \log(L)$. Relative to the one-state GLM, the two-state HMM-GLM yielded a reduction in BIC by $\delta_{BIC} = -1.12 \times 10^3$ (red). We did not observe this reduction when we randomly shuffled the trials for a total of 100 iterations, and computed BICs for these randomly permuted data (average $\delta_{BIC} = -323.8$; blue). As expected, shuffling did not have an effect on BIC in the one-state control GLM.

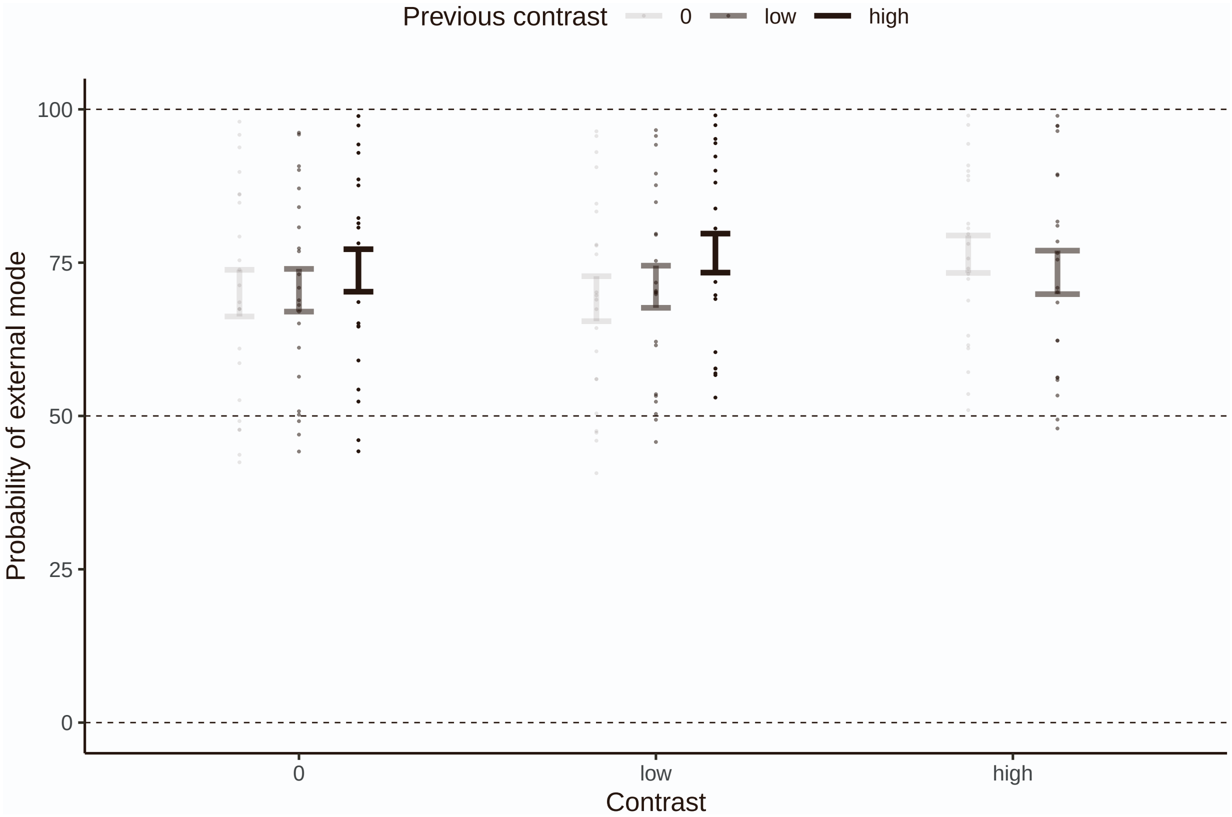

Figure S2. External mode as a function of contrast at the current and the previous trial. Related to Figure 2 and 3. As an alternative explanation for external and internal modes, one may assume a response heuristic that uses the external sensory data when it is reliable (i.e., at high-contrast trials), and applies internal predictions when the external sensory data is not reliable (i.e., no-stimulus and low-contrast trials). The latter explanation would entail that the trial-wise probability of external mode depends exclusively on contrast. We found that the probability of the external mode was higher when the contrast at the current trial was high (0.19 ± 0.03, z = 7.58, $p < 0.001$). Likewise, the external mode was more likely when the contrast of the stimulus presented at the preceding trial was high (0.22 ± 0.03, z = 8.5, $p < 0.001$). This may suggest that stimuli with high signal-to-noise ratio make switches to the external mode more likely. However, the overall change in the probability of external mode was small, ranging form a minimum of 69.12% to a maximum of 76.56%. Moreover, the average interval between mode switches (59.1 ± 8.8 trials) was almost eight times longer than the average interval between high-contrast stimuli

(8.66 $\pm$ 0.06 trials). Our analyses therefore reveal a potential role of high signal-to-noise ratio for mode alternations. They are, however, not compatible with the view that external and internal modes reflect response strategies applied exclusively to stimuli at high and low signal-to-noise ratio, respectively.
